# Assessment of myocardial function in obstructive hypertrophic cardiomyopathy cats with and without response to medical treatment by carvedilol

**DOI:** 10.1186/s12917-019-2141-0

**Published:** 2019-10-28

**Authors:** Ryohei Suzuki, Yohei Mochizuki, Yunosuke Yuchi, Yuyo Yasumura, Takahiro Saito, Takahiro Teshima, Hirotaka Matsumoto, Hidekazu Koyama

**Affiliations:** 1 Laboratory of Veterinary Internal Medicine, Division of Therapeutic Sciences 1, Department of Veterinary Clinical Medicine, Faculty of Veterinary Medicine, Nippon Veterinary and Life Science University, 1-7-1 Kyonan-cho, Musashino-shi, Tokyo, 180-8602 Japan; 2 Faculty of Veterinary Medicine, Okayama University of Science, 1-3 Ikoinooka, Imabari-shi, Ehime 794-8555 Japan

**Keywords:** Beta-blocker, Cat, Echocardiography, Heart, Speckle-tracking, Strain

## Abstract

**Background:**

Inconsistency of treatment response in cats with obstructive hypertrophic cardiomyopathy is well recognized. We hypothesized that the difference in response to beta-blockers may be caused by myocardial functional abnormalities. This study was designed to compare myocardial function in cats with obstructive hypertrophic cardiomyopathy with and without response to beta-blockers. Twenty-one, client-owned, hypertrophic cardiomyopathy cats treated with carvedilol were analyzed. After carvedilol treatment, cats with decreased left ventricular outflow tract velocity were categorized as responders (*n* = 10); those exhibiting no response (no decrease in the left ventricular outflow tract velocity) were categorized as non-responders (*n* = 11). The cats were examined using layer-specific assessment of the myocardial function (whole, endocardial, and epicardial layers) longitudinally and circumferentially by two-dimensional speckle-tracking echocardiography, before and after carvedilol treatment.

**Results:**

The non-responder cats had a significantly higher age, end-diastolic left ventricular posterior-wall thickness, peak velocity of left ventricular outflow tract, and dose of carvedilol than the responders (*p* = 0.04, *p* < 0.01, *p* < 0.01, and *p* < 0.01, respectively). The circumferential strain in the epicardial layer was lower and circumferential endocardial to epicardial strain ratio was higher in non-responders than responders (*p* < 0.001 and *p* = 0.006). According to the multivariate analysis, circumferential strain in the epicardial layer was the only independent correlate of treatment response with carvedilol.

**Conclusions:**

Myocardial function, assessed by two-dimensional speckle-tracking echocardiography, differed in cats with hypertrophic cardiomyopathy with and without response to beta-blockers. The determination of layer-specific myocardial function may facilitate detailed pathophysiologic assessment and treatment response in cats with hypertrophic cardiomyopathy.

## Background

Hypertrophic cardiomyopathy (HCM), a primary disorder of the myocardium, is the most common cardiac disease in cats [1–4]. The REVEAL study demonstrated that preclinical (asymptomatic) cats with HCM have substantial risk for congestive heart failure, arterial thromboembolism, and cardiovascular death [4]. We recently demonstrated that cats with HCM have latent myocardial dysfunction, as assessed by two-dimensional speckle-tracking echocardiography (2D-STE), even in the asymptomatic state [5–8].

Some cats with HCM have dynamic left ventricular outflow tract (LVOT) obstruction [2, 4, 9, 10]; this condition is known as obstructive HCM. The LVOT obstruction causes a pressure overload in the left ventricle and mitral regurgitation due to systolic anterior motion of the mitral valve [11]. These abnormalities may induce further hypertrophy, delayed ventricular relaxation, increased left ventricular (LV) diastolic pressure, myocardial ischemia, and decreased cardiac output [11]. We empirically use beta-blockers (Carvedilol) for the treatment of dynamic LVOT obstruction, so as to block the effects of catecholamines that exacerbate the outflow tract obstruction and to decrease the heart rate so that diastolic filling is enhanced [12]. Although treatment with beta-blockers is generally effective, some cases may prove refractory (peak velocity of LVOT does not decrease). We hypothesized that the inconsistency of response to beta-blockers may be caused by myocardial functional abnormalities. Therefore, the purpose of this study is to compare the myocardial function in cats with and without response to treatment with beta-blockers.

## Results

### Feline profiles and standard echocardiography

The pre-examination (before treatment) characteristic data for cats with obstructive HCM with (Responder) and without (Non-responder) response to medical treatment are summarized in Table 1. Body weight, heart rate, and systolic blood pressure did not differ significantly between responder and non-responder cats. The non-responder cats had a significantly higher age, end-diastolic LV posterior-wall thickness (LVPWd), peak velocity of the LVOT, and dose of carvedilol than the responder cats (*p* = 0.04, *p* < 0.01, *p* < 0.01, and *p* < 0.01, respectively). Standard echocardiographic pre-examination (before treatment) data for cats with obstructive HCM with (Responder) and without (Non-responder) response to medical treatment are summarized in Table 2. All standard echocardiographic variables at pre-examination (before treatment), except for LVPWd and peak velocity of the LVOT, did not differ significantly between responder and non-responder cats.

**Table 1:** Characteristic pre-examination (before treatment) data for cats with obstructive hypertrophic cardiomyopathy

	Responder	Non-responder
Number of cats (male)	10 (5)	11 (9)
Age, months	15.6 (9.6, 22.8)	28.8 (21.6, 52.8)*
Body weight, kg	3.9 (3.4, 4.1)	3.7 (3.0, 4.1)
Heart rate, bpm	216 (186, 222)	196 (184, 232)
Systolic blood pressure, mmHg	126 (111, 143)	125 (113, 134)
Peak velocity of LVOT, m/s	3.7 (3.6, 3.8)	4.6 (4.3, 5.1)*
Carvedilol dose, mg/kg/day	0.2 (0.2, 0.6)	0.6 (0.56, 0.6)*
Medical treatment period, days	107 (78, 273)	161 (77, 256)

Data are expressed as medians (25, 75% interquartile ranges).

LVOT: left ventricular outflow tract.

* Within a row, values differed significantly (*p* < 0.05) from Responder

**Table 2:** Standard echocardiographic pre-examination (before treatment) data for cats with obstructive hypertrophic cardiomyopathy

	Responder	Non-responder
LA/Ao	1.3 (1.2, 1.4)	1.5 (1.4, 1.6)
IVSd, mm	6.1 (5.9, 6.8)	7.2 (6.5, 7.6)
LVPWd, mm	4.9 (3.9, 5.9)	6.6 (6.3, 8.3) *
LVIDd, mm	14.3 (13.5, 16.0)	13.1 (10.6, 14.5)
LVIDs, mm	6.9 (3.0, 9.8)	6.0 (4.5, 6.6)
FS, %	50.0 (42.9, 57.2)	54.9 (51.7, 61.2)
E wave velocity, m/s	1.1 (0.9, 1.2)	0.8 (0.8, 1.0)
Deceleration time of E wave, ms	56.2 (50.2, 75.4)	50.6 (42.6, 71.8)
A wave velocity, m/s	1.0 (0.9, 1.1)	1.0 (0.9, 1.1)
E/A ratio	0.9 (0.9, 1.1)	0.8 (0.7, 0.9)

Data are expressed as medians (25, 75% interquartile ranges).

IVSd: end-diastolic interventricular septal thickness; FS: fractional shortening; LA/Ao: left atrial to aortic root ratio; LVIDd: end-diastolic left ventricular internal diameter; LVIDs: end-systolic left ventricular internal diameter; LVPWd: end-diastolic left ventricular posterior-wall thickness.

*Within a row, values differed significantly (*p* < 0.05) from Responder

After medical treatment, the peak velocity of LVOT was significantly decreased compared to before treatment values in responder cats (1.3 [1.0, 1.4] m/s, *p* < 0.01), but did not change in non-responder cats (4.2 [3.6, 5.0] m/s). The heart rate was significantly decreased in both responder (155 [149, 173] m/s, *p* < 0.01) and non-responder (167 [151, 181] m/s, *p* < 0.01) cats. In responder cats, end-diastolic interventricular septal thickness (IVSd) was decreased at post-examination (167 [151, 181] mm, *p* = 0.04). In non-responder cats, end-diastolic LV internal diameter (LVIDd) and end-systolic LV internal diameter (LVIDs) were increased at post-examination (LVIDd, 14.7 [12.8, 15.3] mm, *p* = 0.04; LVIDs, 6.3 [5.6, 8.3] mm, *p* = 0.01). LVPWd and fractional shortening (FS) in non-responder cats were decreased at post-examination (LVPWd, 6.1 [5.8, 7.6] mm, *p* = 0.01; FS, 48.3 [43.7, 56.2], *p* = 0.03).

### 2D-STE

The average standard deviation and average coefficients of variation for intra-observer and inter-observer reliability for layer-specific longitudinal and circumferential strains are summarized in Table 3.

**Table 3:** Intra- and Inter-observer measurement variability for layer-specific two-dimensional speckle-tracking echocardiography

	Intra-observer		Inter-observer	
	SD	CV (%)	SD	CV (%)
Longitudinal strain				
Whole layer	0.67	4.3	0.85	5.6
Endocardial layer	0.76	3.9	1.04	6.0
Epicardial layer	0.73	5.8	0.74	5.8
Endo/Epi	0.04	3.1	0.02	5.8
Circumferential strain				
Whole layer	0.68	4.0	1.47	7.8
Endocardial layer	1.16	3.6	2.21	7.2
Epicardial layer	0.39	5.2	0.49	5.5
Endo/Epi	0.25	5.6	0.32	8.1

SD: standard deviation; CV: coefficient of variation; Endo/Epi: endocardial to epicardial ratio.

The global 2D-STE data for cats with obstructive HCM with (Responder) and without (Non-responder) response to medical treatment are summarized in Table 4. The global circumferential strain in the epicardial layer was significantly lower in non-responder cats than in responder cats (*p* < 0.001). Furthermore, the circumferential endocardial to epicardial strain ratio was significantly greater in non-responder than responder cats (*p* = 0.006). However, there was no significant difference between responder and non-responder cats with regard to the global longitudinal strains in all layers and the circumferential strains in the whole and endocardial layers.

**Table 4:** Speckle-tracking echocardiographic pre-examination (before treatment) data for cats with obstructive hypertrophic cardiomyopathy

	Responder	Non-responder
Global longitudinal strain		
Whole layer, %	−15.8 (−11.2, −16.9)	−13.3 (−11.5, −14.2)
Endocardial layer, %s	−18.5 (− 12.9, − 19.7)	−16.3 (− 13.5, − 16.4)
Epicardial layer, %	−13.5 (− 9.8, − 14.7)	−10.6 (− 9.9, − 12.0)
Endo/Epi	1.4 (1.3, 1.4)	1.3 (1.3, 1.4)
Global circumferential strain		
Whole layer, %	−18.3 (− 13.6, − 20.7)	− 15.5 (− 12.9, − 17.2)
Endocardial layer, %	− 31.7 (− 21.6, − 39.0)	− 29.4 (− 26.1, − 33.0)
Epicardial layer, %	−8.7 (− 8.3, − 9.6)	−5.3 (− 4.8, − 6.7) *
Endo/Epi	3.4 (3.0, 3.9)	4.6 (4.0, 6.6) *

Data are expressed as medians (25, 75% interquartile ranges).

Endo/Epi: endocardial to epicardial ratio.

*Within a row, values differed significantly (*p* < 0.05) from Responder

Results of the receiver operating characteristic (ROC) curves and the respective area under the ROC curve (AUC) values to assess the comparative accuracy for identifying cats without response to medical treatment are shown in Table 5. Univariate logistic regression analyses showed that LVPWd, circumferential strain in the epicardial layer, and circumferential endocardial to epicardial strain ratio were significantly related to treatment response. Subsequently, multivariate analysis identified circumferential strain in the epicardial layer as the only independent correlate to detect the variable for identifying cats without response to medical treatment (Table 6).

**Table 5:** Results of the receiver operating characteristic curves to assess the accuracy for identifying non-responders

	Cut off	AUC	Sensitivity	Specificity
LVPWd, mm	6.2	0.86	0.75	0.78
Global circumferential strain				
Epicardial layer, %	−6.85	0.86	0.82	0.9
Endo/Epi	3.9	0.87	0.91	0.8

AUC: area under the receiver operating characteristic curve; Endo/Epi: endocardial to epicardial ratio; LVPWd: end-diastolic left ventricular posterior-wall thickness.

**Table 6:** Results of logistic regression analysis to detect the variable for identifying non-responders

	Univariate analysis	*p* value	Multivariate analysis	*p* value
	Odds ratio (95% CI)		Odds ratio (95% CI)	
LVPWd	4.92 (1.21–19.92)	0.0256		
Global circumferential strain				
Epicardial layer	2.48 (1.17–5.24)	0.0178	2.53 (1.07–5.24)	0.0342
Endo/Epi	5.89 (1.01–34.14)	0.0481		

CI: confidence interval; Endo/Epi: endocardial to epicardial ratio; LVPWd: end-diastolic left ventricular posterior-wall thickness.

## Discussion

In the present study, the heart rate was significantly decreased after carvedilol medical treatment in both responder and non-responder cats. Carvedilol is a non-selective beta-adrenergic receptor blocker and provides negative chronotropic effect in these cats [13, 14]. LV internal dimension, wall thickness, and FS were also changed after treatment. These changes would be caused by increased LV volume due to prolonged diastolic phase and negative inotropic effect of carvedilol [13, 14]. Although these changes were statistically significant, all variables were within the acceptable range, and none of the included cats developed adverse effects after carvedilol treatment. Conversely, carvedilol itself affects the cardiac function in both responder and non-responder cats. The difference in response to carvedilol treatment between responder and non-responder cats would not be caused by the difference of susceptibility and bioavailability of carvedilol in these cats.

Circumferential strain in epicardial layer was lower in non-responder cats than responder cats, and it was most associated with LVOT reduction by medical treatment. As the epicardial myocardium may not be affected by passive contraction unlike the endocardial myocardium, epicardial strain could reflect truly contraction in the myocardium [8, 15]. Histopathologically, myocardial hypertrophy, fiber disarray, intramural coronary arterial narrowing, myocardial ischemia, and fibrosis developed in the myocardium of cats with HCM [16]. These myocardial changes may deteriorate the epicardial contraction [8, 15]. Lower circumferential strain in the epicardial layer may reflect more severe myocardial histopathological changes in non-responder cats and cause the lack of response to medical treatment.

In contrast, higher circumferential endocardial to epicardial strain ratio was observed in non-responder than responder cats. Increased circumferential endocardial to epicardial strain ratio may reflect circumferential endocardial compensation for depressed epicardial contraction [8, 15, 17]. This compensation may contribute to prevent the development of cardiac symptoms in these cats.

The peak velocity of LVOT at baseline (before treatment) was higher in non-responder cats than responder cats. The LVOT obstruction causes a pressure overload in the left ventricle and mitral regurgitation due to systolic anterior motion of the mitral valve [11]. As earlier human 2D-STE studies have demonstrated that LVOT obstruction deteriorates the myocardial function in patients with HCM [18], baseline severity of LVOT obstruction may be related to the treatment response. Nevertheless, multivariate regression analysis showed that circumferential strain in the epicardial layer (myocardial functional variable) was the only predictor of treatment response in this study. In human patients with HCM, deterioration of myocardial strain was demonstrated as an independent predictor of adverse outcomes [19, 20]. Although clinical outcome was not assessed in this study, non-responder cats continued to have cardiac load of LVOT obstruction and may have had adverse outcomes. Therefore, the evaluation of myocardial function has the potential to allow early identification of cats at risk for progression to symptomatic and severe HCM. The clinical importance of myocardial function using 2D-STE and their relationship to survival time needs to be further investigated.

Baseline LVPWd was also higher in non-responder cats than responder cats. The degree of myocardial histopathological changes and severity of wall thickness were correlated [21]. The relationship between the amount of myocardial fibrosis and speckle-tracking derived strain variables has been demonstrated [21, 22]. Although histopathological characteristics were not assessed in this study, layer-specific myocardial strains might provide additional insights regarding the extent and distribution of myocardial changes.

In the present study, there was no significant difference in the global longitudinal strains in all layers between responder and non-responder cats. Previous feline studies have demonstrated that longitudinal function was depressed in cats with obstructive HCM compared to normal cats [6–8, 23]. Although there was no significant difference between the responder and non-responder cats, longitudinal strains in both the responder and non-responder group were as low as in earlier studies including cats with obstructive HCM [6–8, 23]. Therefore, longitudinal strain assessment of the myocardium using 2D-STE may be useful to distinguish between cats with asymptomatic HCM and healthy cats. In cats with obstructive HCM, the myocardial function differs based on the myocardial contractile direction and the layer of the myocardium. Therefore, the determination of multidirectional and layer-specific myocardial function may facilitate a detailed assessment of systolic function in cats with HCM.

This study has several limitations. First, the ages of the responder and non-responder cats varied. Age difference between groups may affect the treatment responses by carvedilol. However, an earlier 2D-STE study had demonstrated that systolic myocardial strains were less affected by aging [24]. Second, because our study was a non-invasive clinical investigation, we could not definitively measure LV load by using cardiac catheterization. Thus, we did not assess the LV functional improvement by medical treatment in responder cats. Third, definitive diagnosis and myocardial changes of the included cats were not confirmed by the histopathological examinations. Some cats with HCM, however, were misdiagnosed in this study. Forth, high-quality B-mode images for the 2D-STE analysis are sometimes difficult to obtain, especially in the feline heart. However, 2D-STE variables including layer-specific global strains had adequate measurement variability in this study. Finally, the number of our cats was small, which may affect the statistical power and may limit the extrapolation of these findings to larger populations. However, detailed and precise myocardial functional data were used in this study. These limitations should be overcome in future studies.

## Conclusions

Myocardial function assessed by 2D-STE, differed in cats with obstructive HCM with and without response to medical treatment. Baseline LVPWd, peak velocity of LVOT, circumferential strain in the epicardial layer, and circumferential endocardial to epicardial strain ratio were related to LVOT reduction after carvedilol treatment. According to the multivariate analysis, circumferential strain in the epicardial layer was the only predictor of treatment response. Lower circumferential strain in the epicardial layer may reflect more severe myocardial histopathological changes in non-responder cats and may cause the lack of response to medical treatment. Measurement of myocardial function using 2D-STE may provide a more detailed assessment of contractile function and treatment response in cats with HCM. Hence, the clinical importance of these strains and their relationship to survival time requires further investigation.

## Methods

### Animals

We reviewed the medical records of 33 client-owned cats that were diagnosed with HCM and treated with carvedilol (Artist, Daiichi Sankyo Co. Ltd., Tokyo, Japan) at the Veterinary Medical Teaching Hospital of the Nippon Veterinary and Life Science University. These cats were presented for cardiac screening because of cardiac murmur or suspected cardiac abnormalities by a referral hospital during the period from October 2010 to April 2018, and their clinical findings were analyzed retrospectively. We diagnosed HCM with echocardiographic evidence of the LV hypertrophy and absence of the other diseases known to cause LV hypertrophy. Echocardiographic LV hypertrophy was judged if cats with the LV wall thickness at end-diastole was 6 mm or more, as measured by two-dimensional methods. LV thickness was measured from the short-axis view, and the mean values of 3 consecutive cardiac cycles of the thickest segment were used. We excluded cats that had systolic blood pressure > 160 mmHg (non-invasive oscillometric method) or systemic and other cardiovascular diseases, including dehydration. Three cats were excluded from this study because they were treated with other medications that affected hemodynamics (i.e., atenolol or diltiazem) at the initial examination; nine other cats were excluded because they did not complete follow-up examinations. Finally, we included 21 cats with obstructive HCM in this study. The breeds of HCM cats were Scottish fold (*n* = 7), Domestic cat (*n* = 3), Maine coon (n = 3), American shorthair (*n* = 2), Exotic shorthair (*n* = 1), Munchkin (n = 1), Norwegian forest cat (n = 1), Ragdoll (n = 1), Russian blue (n = 1), and Sphinx (n = 1). All cats with HCM had echocardiographic evidence of dynamic LVOT obstruction and systolic anterior motion of the mitral valve, in this study. LVOT obstruction was defined as turbulent LV outflow with high velocity (> 2.5 m/s) using continuous-wave Doppler ultrasound [4, 10, 12]. None of the cats HCM had a history or clinical signs of cardiac disease.

Physical examination, electrocardiogram, thoracic radiography, blood pressure measurement, and echocardiography were performed before (baseline) and after administration of carvedilol. All cats were divided into 2 groups depends on the treatment responses; responder group (*n* = 10, peak velocity of LVOT was decreased < 2.5 m/s on post-examination) and non-responder group (*n* = 11, peak velocity of LVOT was maintained > 2.5 m/s on post-examination). Carvedilol doses were gradually increased until the target dose was reached, or until systolic cardiac murmur disappeared. Target carvedilol doses were set above 0.4 mg/kg/day to achieve the beta-adrenergic stimulus blockade in cats [25]. Post-examination data after carvedilol treatment were acquired at least 1 week after the target carvedilol dose had been reached for each cat.

### Standard echocardiography

Conventional two-dimensional and Doppler examinations were performed using an echocardiographic system (Vivid 7 and Vivid E95, GE Healthcare, Tokyo, Japan); the simultaneous ECG limb lead II was recorded and displayed on the images. All data were obtained for at least 3 consecutive cardiac cycles in sinus rhythm from non-sedated cats that were manually restrained in the right and left lateral recumbent positions. A single trained observer analyzed the images using an off-line workstation (EchoPAC PC-version 201, GE Healthcare, Tokyo, Japan). The observer was not a member of the cardiology team that performed echocardiography and he is blinded to group allocation during the analysis. The left atrial to aortic root ratio was obtained from the right parasternal short-axis view using the B-mode method [26]. The IVSd, LVPWd, LVIDd, LVIDs, and FS were measured using the B-mode method from the right parasternal short-axis view of the LV. Transmitral flow was evaluated from the left apical 4-chamber view, and the peak velocity of the early diastolic wave (E wave), deceleration time of the E wave, and peak velocity of the late diastolic wave (A wave) were measured. In cats with fusion of the E and A waves, values for those waves were not used. For all analyses, the mean values of 3 consecutive cardiac cycles in sinus rhythm from high-quality images were used.

### Two-dimensional speckle-tracking echocardiography

**Fig. 1:**
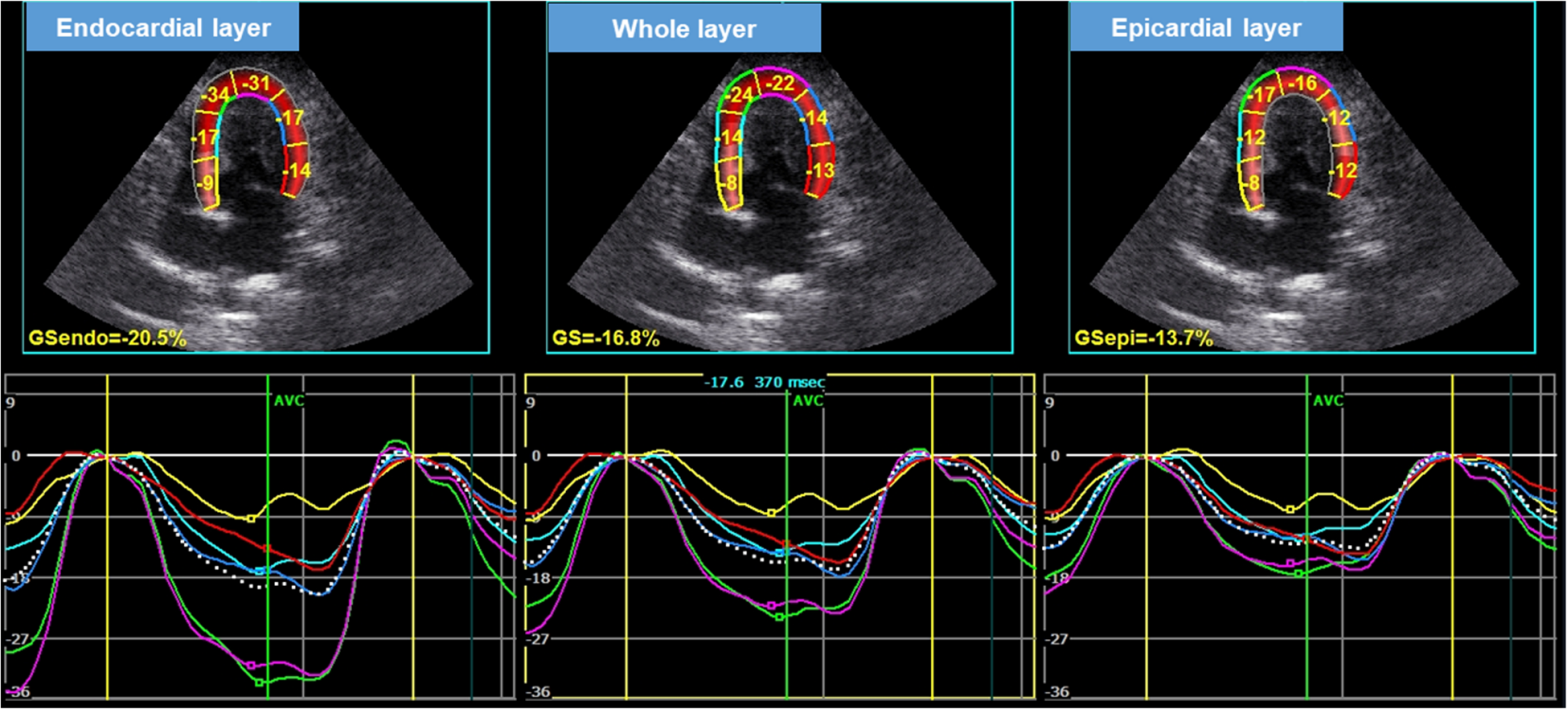
Layer-specific (whole, endocardial, and epicardial layer) longitudinal strain curves in a cat with hypertrophic cardiomyopathy. Dotted line is indicated global strain curve obtained from two-dimensional speckle-tracking echocardiography (left apical four-chamber view). Six segmental curves (colored lines) are designated as the basal septum (yellow), middle septum (light blue), apical septum (green), apical lateral (purple), middle lateral (dark blue), and basal lateral (red) for speckle tracking analysis.

**Fig. 2:**
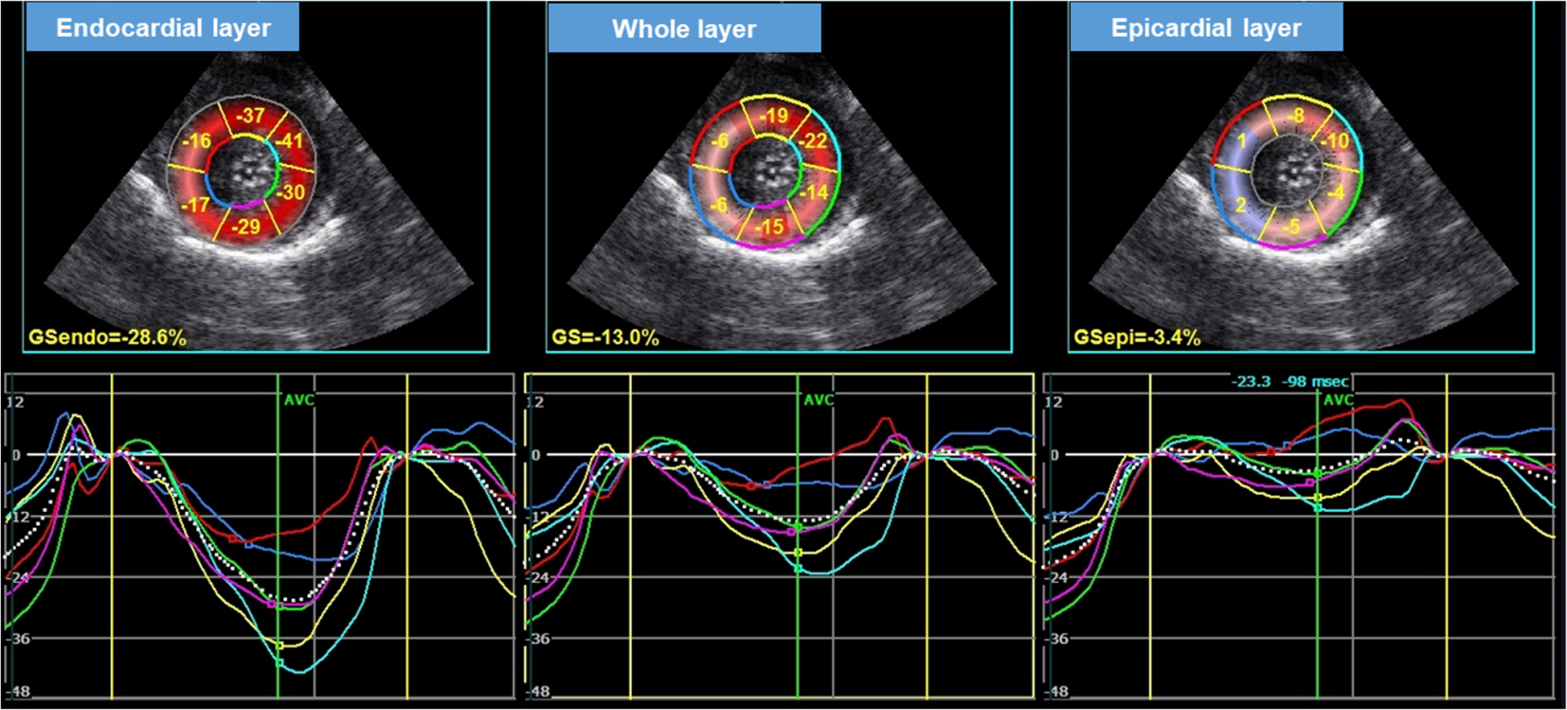
Layer-specific (whole, endocardial, and epicardial layer) circumferential strain curves in a cat with hypertrophic cardiomyopathy. Dotted line is indicated global strain curve obtained from two-dimensional speckle-tracking echocardiography (right parasternal short-axis view). Six segmental curves (colored lines) are designated as the cranial septum (yellow), cranial (light blue), lateral (green), caudal (purple), inferior (dark blue), and septum (red) for speckle tracking analysis.

Two-dimensional speckle-tracking echocardiography protocols that were previously published for dogs [24, 27–30] and cats [5–8] were applied. High-quality images for 2D-STE analysis were carefully obtained by the same investigator. To evaluate circumferential deformation by 2D-STE, we used a right parasternal short-axis view of the LV at the level of the papillary muscles. A left apical 4-chamber view was used to analyze longitudinal deformation. Images were again analyzed by a single observer using an offline workstation (EchoPAC PC-version 201, GE Healthcare, Tokyo, Japan), as described previously [5–8, 24, 27–30]. The observer was not a member of the cardiology team that performed echocardiography and he is blinded to group allocation during the analysis. Our protocols for 2D-STE analysis in cats were described previously [5–8]. We measured the peak systolic strain of the endocardial, whole, and epicardial layers in the longitudinal (Fig. 1) and circumferential directions (Fig. 2). We also calculated the endocardial to epicardial strain ratio, which may reflect endocardial compensation of the myocardium [8, 15, 17]. The mean values of the measurements from 3 consecutive cardiac cycles using high-quality images were used in all analyses.

### Statistical analysis

Data are expressed as medians and interquartile ranges. Shapiro-Wilk test was used to check the normal distribution of the variables. The Wilcoxon signed-rank test was used to compare the pre-examination (before treatment) with post-examination (after treatment) variables. Because of the most variables are non-parametric data, Mann-Whitney *U-*test was used to compare the before treatment variables between responder and non-responder cats. To assess the comparative accuracy of different echocardiography variables for identifying cats without response to medical treatment, ROC curves and the respective AUC were calculated for the pre-examination variables, with the level of significance set at *p* < 0.05 in the Mann-Whitney *U-*test. Cut-off value was determined based on the minimum distance to the upper left corner in each ROC curve (Youden’s index). Predictors of treatment response were assessed by logistic regression analysis. Echocardiographic variables at the pre-examination with *p* < 0.05 in univariate analyses were included in the multivariate analysis, which was performed by the stepwise method. Values of *p* < 0.05 were considered significant. All statistical analyses were performed with appropriate statistical software (R software version 2.8.1., The R Foundation for Statistical Computing, Vienna, Austria).

Intra-observer reproducibility was assessed by determining the coefficients of variation by having the observer repeat the measurements three times for three cats selected at random on different days. The studies were also analyzed by a second blinded observer to assess inter-observer reproducibility. The second observer was not a member of the cardiology team that performed echocardiography and had not known to the results of echocardiography by first observer.

## Data Availability

The datasets used and/or analyzed during the current study are available from the corresponding author on reasonable request.

## Abbreviations

2D-STE: Two-dimensional speckle-tracking echocardiography
AUC: Area under the receiver operating characteristic curve
FS: Fractional shortening
HCM: Hypertrophic cardiomyopathy
IVSd: End-diastolic interventricular septal thickness
LV: Left ventricular
LVIDd: End-diastolic left ventricular internal diameter
LVIDs: End-systolic left ventricular internal diameter
LVOT: Left ventricular outflow tract
LVPWd: End-diastolic left ventricular posterior-wall thickness
ROC: Receiver operating characteristic
